# Digital inclusion in China’s national smart health and older adult care policy design: an access-oriented and uneven embedding pattern, 2013–2025

**DOI:** 10.3389/fpubh.2026.1944635

**Published:** 2026-09-29

**Authors:** Haiyan Wu, Miao Xing, Yunhan Wang, Yu Jia, Qinming Yu

**Affiliations:** 1 College of Humanities and Management, Heilongjiang University of Chinese Medicine, Harbin, China; 2 First Affiliated Hospital of Heilongjiang University of Chinese Medicine, Harbin, China

**Keywords:** China, content analysis, digital inclusion, healthy ageing, older adults, policy instruments, smart health and older adult care

## Abstract

**Background:**

Population ageing and rapid digitalisation of health and care services have raised concerns about whether older adults are adequately considered in digital policy design. Although digital inclusion is an increasingly visible policy goal, little is known about how national policy instruments embed digital inclusion or respond to older adults’ multidimensional needs.

**Methods:**

We developed a Policy Alignment Framework linking policy instruments, digital inclusion orientations, and older adults’ needs. A quantitative content analysis was conducted on 35 national-level smart health and older adult care policy documents issued in China between 2013 and 2025. In total, 1,033 policy statements were coded and analysed using descriptive statistics, cross-tabulation, Pearson’s chi-square tests, Cramer’s V, and adjusted standardised residuals.

**Results:**

Digital inclusion was structured but unevenly embedded in national policy design. Among all coded statements, 48.8% contained no explicit digital inclusion orientation, 42.2% addressed access and environment, and only 9.0% addressed capability-building and safety protections. Policy instruments were significantly associated with digital inclusion orientations. Technology/data and platform interconnection instruments were over-represented in access- and environment-oriented inclusion, while capability-building and safety-protection measures were mainly concentrated in fiscal and regulatory instruments. Older adults’ needs were also unevenly aligned with digital inclusion. Health care and service access needs dominated the policy corpus, whereas social–emotional support, development and autonomy, and participation-oriented needs received limited attention. Overall, digital inclusion was framed mainly as infrastructure, platform development, and service accessibility rather than a broader capability, affordability, safety, and participation challenge.

**Conclusion:**

This study identifies an access-oriented and uneven embedding pattern of digital inclusion across policy instruments and need domains in China’s national smart health and older adult care policy design. Moving towards equity-sensitive digital ageing policy requires greater attention to capability-building, affordability support, safety protection, offline fallback arrangements, and participation-oriented support. The Policy Alignment Framework offers a transferable tool for assessing digital inclusion governance in ageing and digitally transforming societies. As this study examines national policy design rather than implementation outcomes, the findings indicate policy intent and textual orientation, not actual digital inclusion effects.

## 1 Introduction

Population ageing and digital transformation are reshaping public service delivery worldwide, and China illustrates this convergence in acute form. According to the National Bureau of Statistics of China, by the end of 2025, China had 323.38 million people aged 60 years and older, accounting for 23.0% of the population, and 223.65 million people aged 65 years and older, accounting for 15.9% (1). In parallel, services ranging from appointment booking and chronic-disease management to emergency response and welfare access have increasingly shifted online, often adopting a “digital by default” logic (2, 3). For many users, this transition may improve convenience and service efficiency. For older adults, however, it may create a compounding risk: those who cannot navigate digital systems may be excluded from the very services intended to support their health, care, and social participation. The intersection of demographic ageing and technological restructuring has therefore become a pressing policy-design challenge in China and in ageing societies more broadly.

Research on the digital divide has increasingly moved beyond a simple access-based understanding toward a multidimensional view of digital inequality. The first-level divide concerns access to devices and connectivity; the second-level divide concerns digital skills and patterns of use; and the third-level divide concerns the unequal ability to convert digital engagement into meaningful health, social, and economic outcomes (3–5). In response, digital inclusion has been conceptualised as a broader, rights-based idea: not access alone, but sustained, meaningful, affordable, and safe participation in digital environments regardless of age, income, education, or place of residence (6, 7). For older adults, inclusion in this sense spans infrastructure access, digital skills, affordability, online safety, offline fallback arrangements, and the protection of digital rights. It is therefore a multidimensional equity issue with clear public health relevance, requiring a multidimensional policy response.

Digital inclusion does not operate independently of the governance context that produces it. In health and care services for older adults, it is shaped by the instruments governments choose, because policy instruments translate broad policy goals into operational measures and influence how resources, obligations, and services are distributed (8, 9). We therefore examine inclusion along three dimensions. The first is policy instruments—the tools through which policy goals become action, from technology deployment and platform construction to fiscal support, service provision, and regulation. The second is digital inclusion orientations—how instruments embed inclusion, whether through expanded access and age-friendly environments, through capability support and safeguards, or through no explicit inclusion orientation. The third is older adults’ needs—the range of health, participation, security, autonomy, and social support needs that inclusion policy is expected to meet. In this study, “smart health and older adult care” refers to the Chinese policy field often translated as “smart health and elderly care”; age-inclusive language is used except when referring to official policy titles or search terms. Together, these dimensions form the analytical architecture of this study and allow us to examine how China’s national smart health and older adult care policy texts connect digital tools to the needs of an ageing population.

Making sense of older adults’ needs requires a theoretically grounded account of what those needs are. We use the World Health Organization’s Active Ageing Policy Framework as the foundation for this study (10). The framework organises later-life needs into three interdependent pillars: health, including physical and mental well-being and access to care; participation, including social engagement, belonging, and intergenerational connection; and security, including economic security, safety, rights, and dignity. It also emphasises older adults’ agency, autonomy, learning, and contribution as important dimensions of ageing policy. Drawing on this framework and prior needs classifications in the Chinese ageing-policy context, we operationalise these pillars into six functional domains: health care, service access and environment, rights and safety, economic support, social–emotional support, and development and autonomy (11).

Whether policy instruments are textually matched to these needs—and whether they extend inclusion beyond access to capability, affordability, safety, and offline fallback support—is a policy-design question with relevance for digital health equity. It has received limited systematic attention in China. Existing studies have examined individual-level drivers of digital exclusion, such as education, income, digital skills, and health literacy, or have described the evolution of smart health and older adult care policies (5, 12–14). Useful as these studies are, they leave a basic policy-design question open: how are the tools specified in China’s national smart health and older adult care policy texts associated with the layered needs of older adults, and how is inclusion textually embedded in relation to the three-level digital divide? The closest prior work, Zeng and Han (14), analysed 305 policy statements from 34 documents issued up to December 2021 using a three-dimensional framework of policy instruments, digital divide levels, and actors. The present study extends this line of inquiry in three respects. First, it covers a longer and more recent period, from 2013 to 2025. Second, it codes a larger statement-level corpus of 1,033 statements from 35 documents. Third, it replaces the actor dimension with a needs dimension grounded in the WHO Active Ageing Framework. This shifts the analytical focus from who implements policy to whose needs the policy addresses. It also moves beyond descriptive frequency counts to examine textual association patterns among dimensions using chi-square analysis and adjusted standardised residuals.

Three gaps motivate this study. First, existing policy analyses have largely retained a digital divide framing centred on access rather than the broader rights-based inclusion perspective now common in health-equity scholarship (6, 7). As a result, capability-building, affordability, safety, and offline fallback arrangements have rarely been treated as design concerns in their own right. Second, prior needs classifications have tended either to remain close to service-delivery categories or to be insufficiently linked to an internationally recognised healthy-ageing framework such as the WHO model. Third, no study has tested the textual association patterns among policy instruments, inclusion orientations, and older adults’ needs within a single framework, so the alignment question remains empirically open at the level of national policy design.

To address these gaps, we build and apply an integrated Policy Alignment Framework that places policy instruments, inclusion orientations, and older adults’ needs in a single cross-tabulation structure. Using quantitative content analysis of 35 national policy documents issued between 2013 and 2025, we code 1,033 policy statements across the three dimensions and examine textual association patterns and change over time.

This study does not assess the implementation effectiveness of smart health and older adult care policies, nor does it evaluate their direct effects on health outcomes. Instead, it focuses on national-level policy design and examines how digital inclusion is textually embedded in China’s national smart health and older adult care policy framework. Accordingly, the unit of inference is the policy-design orientation reflected in national policy statements, rather than the actual level of digital inclusion experienced by older adults in local service settings.

Specifically, we ask the following research questions:

*RQ1:* To what extent is digital inclusion explicitly embedded in China’s national-level smart health and older adult care policy statements?

*RQ2:* How are different policy instruments associated with digital inclusion orientations in national policy design?

*RQ3:* How are policy instruments textually aligned with different domains of older adults’ needs?

*RQ4:* How are digital inclusion orientations associated with older adults’ need domains in national policy texts, and where are textual misalignments most evident?

The study makes three contributions. First, it provides a large-scale mapping of digital inclusion in China’s national smart health and older adult care policy texts, shifting attention from individual-level digital divide factors to policy design. Second, it develops a needs-based Policy Alignment Framework grounded in the WHO Active Ageing Framework, enabling policy instruments, inclusion orientations, and older adults’ needs to be analysed jointly. Third, it identifies an access-oriented and uneven textual embedding pattern of digital inclusion in national policy design. In this pattern, service supply, platform development, and health care access receive greater textual attention than capability-building, affordability support, safety protections, offline fallback arrangements, and participation-oriented needs.

## 2 Materials and methods

### 2.1 Study design and analytical framework

This study employed a quantitative content analysis design to examine how digital inclusion is textually embedded in China’s national smart health and older adult care policy documents from 2013 to 2025. Quantitative content analysis is appropriate for systematically transforming policy texts into coded categorical data and identifying distributional patterns and textual association patterns (15, 16). The analysis focused on policy design as represented in national policy texts, rather than on policy implementation, service quality, or health outcomes.

To guide coding and interpretation, this study developed a Policy Alignment Framework linking three analytical dimensions: policy instruments, digital inclusion orientations, and older adults’ multidimensional needs. Policy instruments capture the governmental tools used to promote smart health and older adult care; digital inclusion orientations capture whether and how policy statements address older adults’ access to, use of, and protection in digital services; and older adults’ needs capture how health, care, security, participation, and autonomy-related needs are represented in policy texts. Figure 1 presents the Policy Alignment Framework, including the three analytical dimensions, their main categories, and the cross-tabulation logic used in the analysis.

**Figure 1:**
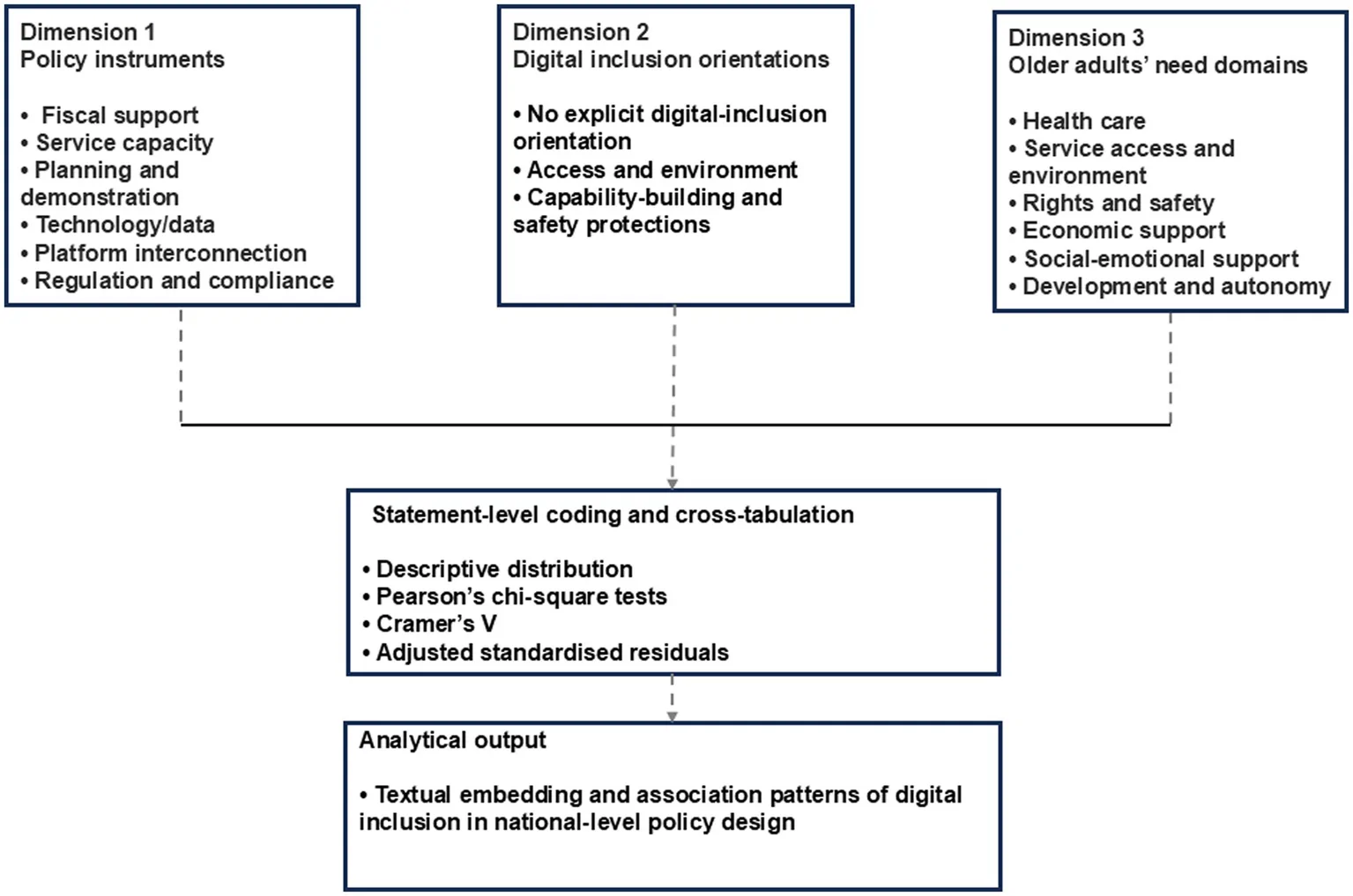
Policy alignment framework. The framework links three analytical dimensions: policy instruments, digital inclusion orientations, and older adults’ need domains. Cross-tabulations were used to examine textual co-occurrence and distributional correspondence among these dimensions.

In this study, policy alignment refers to the textual co-occurrence and distributional correspondence among policy instruments, digital inclusion orientations, and older adults’ needs within policy design. By cross-analysing these three dimensions, the framework assesses whether digital transformation in smart health and older adult care policy is accompanied by inclusion-oriented design and whether policy measures are textually linked to older adults’ multidimensional needs. The study therefore analyses structural emphasis and textual associations in policy design, rather than causal effects or implementation outcomes.

Offline fallback arrangements were not conceptualised as digital participation itself. Rather, they were treated as safety-net mechanisms within a rights-based understanding of digital inclusion, because digital-by-default service delivery may exclude older adults when digital access, skills, affordability, or safety cannot be guaranteed. In this sense, offline fallback arrangements indicate whether policy design recognises the need to prevent exclusion from essential services during digital transformation.

Because policy instruments and need domains may display semantic proximity in policy texts, the framework is used to map textual coupling rather than to infer causal alignment. Instrument categories identify the governmental means specified in a statement, whereas need domains identify the beneficiary problem or need addressed by that statement. This distinction guided the coding procedure and the interpretation of cross-tabulation results.

### 2.2 Data sources and policy document selection

This study focused on national-level policy documents related to smart health and older adult care in China issued between 2013 and 2025. The year 2013 was selected as the starting point because it marked the emergence of national-level policy attention to modernising older adult care services and expanding technology-enabled public service delivery. National-level documents were selected because this study aims to examine central policy-design orientations, agenda-setting priorities, and the formal policy instrument mix articulated by central authorities. These documents provide the national policy framework within which subnational governments formulate and implement more detailed measures, but they do not capture the full range of provincial, municipal, or grassroots adaptations. In China, the official policy term “smart health and elderly care” is commonly used to refer to technology-enabled health and care services for older adults. To follow age-inclusive language conventions, this study uses “smart health and older adult care” except when referring to official policy titles, Chinese search terms, or established translations. Policy documents were collected from official government websites, including the State Council, the General Office of the State Council, the Ministry of Civil Affairs, the Ministry of Industry and Information Technology, the National Health Commission, the National Development and Reform Commission, and other relevant central ministries and commissions. Searches were conducted up to 31 December 2025.

The search covered policy themes related to smart health and older adult care, digital care services for older adults, integrated medical and care services, age-friendly digital transformation, digital inclusion of older adults, the silver economy, health service digitalisation, Internet Plus public services, intelligent care products for older adults, and the smart management of care institutions for older adults. Search terms included Chinese keywords such as “智慧健康养老” (smart health and elderly care), “智慧养老” (smart care for older adults), “互联网 + 养老” (Internet Plus care for older adults), “医养结合” (integrated medical and care services), “适老化改造” (age-friendly transformation), “老年人数字鸿沟” (digital divide among older adults), and “银发经济” (silver economy).

All searches were conducted in Chinese. Candidate documents were first screened by title and issuing authority, and then assessed through full-text reading against the inclusion and exclusion criteria. Documents were included if they met the following criteria: (1) issued by the State Council, national ministries and commissions, or other central government agencies; (2) publicly available formal policy documents issued between 2013 and 2025; (3) containing substantive policy objectives, implementation tasks, governance measures, technical guidelines, standards, catalogues, or institutional arrangements; and (4) directly relevant to older adults or older adult care, and substantively related to health care, digital technologies, age-friendly transformation, intelligent products, or smart service applications.

Documents were excluded if they were news reports, policy interpretations, speeches, local-level policy documents, duplicates, or documents only weakly related to smart health and older adult care. The initial search of government websites identified 37 candidate records. These records were independently screened by the second and third authors, and the first author subsequently reviewed the screening results and cross-checked the candidate list against relevant academic literature indexed in CNKI (China National Knowledge Infrastructure). Through this process, 4 duplicate records were removed and 5 records were excluded for not meeting the inclusion criteria, resulting in 28 retained documents. An additional 7 foundational policy documents, identified through citation searching in CNKI, were subsequently incorporated into the sample. This process yielded a final sample of 35 national-level policy documents (D1–D35). The full list of included documents is provided in Supplementary Table S1.

The overall document identification and screening process is summarised in Figure 2, using a PRISMA-style flow diagram adapted to reflect the screening and supplementary search procedures used in this study.

**Figure 2:**
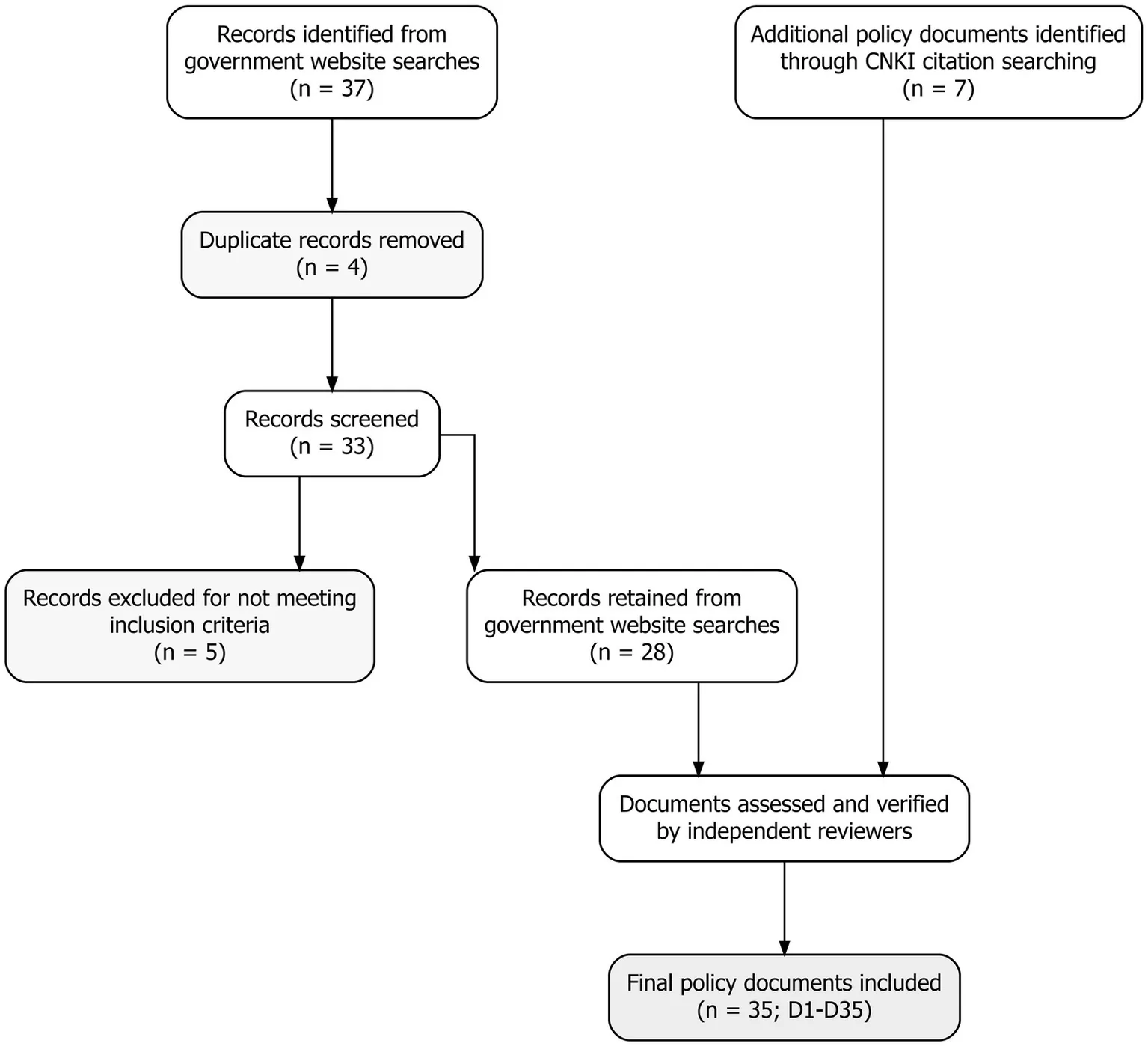
Flow diagram of document identification and screening.

### 2.3 Policy stage classification

To examine temporal shifts in textual policy attention, the 2013–2025 study period was divided into four stages based on publication sequence, major national policy milestones, and thematic shifts in China’s smart health and older adult care policy agenda. The four stages were: policy incubation (2013–2016), pilot access (2017–2019), age-friendly deepening (2020–2023), and silver economy application (2024–2025). The stages were not designed to have equal duration or equal numbers of policy statements; rather, they were intended to capture major shifts in national policy framing and agenda emphasis.

The policy incubation stage was characterised by foundational policies on older adult care services and Internet Plus public services. The pilot access stage was marked by the release and implementation of the Smart Health and Elderly Care Industry Development Action Plan, whose official title is retained here as a policy name. The age-friendly deepening stage was shaped by national policies addressing the difficulties experienced by older adults in using smart technologies. This stage also coincided with the COVID-19 pandemic, during which health codes, contact-tracing platforms, and online service access made digital exclusion among older adults more visible. The silver economy application stage captured the growing integration of smart health and older adult care into silver economy development and application scenarios.

This stage classification was used as a descriptive temporal framework for analysing shifts in policy attention over time, rather than as a causal periodisation or evidence of a stable policy paradigm. In particular, because the silver economy application stage covers only 2024–2025, findings related to this stage are interpreted as short-term textual signals associated with a recent policy window rather than as evidence of a consolidated long-term trajectory.

### 2.4 Unit of analysis and text segmentation

All policy documents were retrieved, read, and coded in their original Chinese language. Text segmentation and coding were conducted directly on the Chinese source texts, rather than on translated versions, to preserve the administrative and policy-specific terminology embedded in the original documents. Policy documents served as the sampling units, whereas policy statements served as the coding and statistical analysis units. A policy statement was defined as a clause or text fragment with relatively independent policy meaning, such as a policy objective, implementation task, governance measure, technical requirement, service arrangement, or target-group specification. Each coding unit was required to contain one dominant and codable policy meaning.

This unit was selected because a single policy document often contains multiple policy objectives, instruments, target groups, implementation requirements, and governance arrangements. Using the full document as the unit of analysis would therefore obscure fine-grained policy content and textual association patterns among policy instruments, digital inclusion orientations, and older adults’ need domains.

Text segmentation was conducted according to clause structure, syntactic boundaries, and semantic completeness. When one sentence contained multiple distinct policy meanings, it was divided into separate coding units. When a text fragment contained overlapping policy meanings, coders identified the dominant policy meaning according to the immediate textual context and the coding rules for each analytical dimension. This procedure was used to reduce ambiguity and to avoid inferring one coding dimension solely from another. Text fragments were excluded if they consisted only of document titles, tables of contents, appendix descriptions, rhetorical statements, repeated expressions, background descriptions, or content without substantive policy meaning. After text preprocessing, segmentation, and data cleaning, 1,033 valid policy statements were obtained and used as coding and statistical analysis units.

### 2.5 Coding scheme and variable aggregation

Following established procedures for quantitative content analysis, a structured coding scheme was developed to convert policy statements into categorical variables across three analytical dimensions: policy instruments, digital inclusion orientations, and older adults’ need domains (15, 16). Each coding unit was assigned one dominant code in each dimension. When a text fragment contained multiple meanings, coders identified the dominant policy function, digital inclusion orientation, and need representation. Dominance was determined according to the main policy action or requirement expressed in the statement, with reference to the coding manual and the immediate textual context. This approach was used to avoid double counting and to maintain mutually exclusive categories for subsequent statistical analysis. All codes were dimension-specific and were interpreted within their respective coding dimensions. Therefore, a code in one dimension was not automatically inferred from a code in another dimension. Illustrative examples of original coding across the three analytical dimensions are provided in Supplementary Table S3.

The policy instrument dimension was coded at two levels, drawing on policy instrument theory and the supply-side, environment-side, and demand-side typology (8, 9). Policy instruments were coded according to the primary governmental means or action mechanism expressed in each statement. The first-level categories included supply-side, environment-side, and demand-side instruments. At the second level, 26 instrument categories were identified, covering service and product provision, platform and infrastructure construction, technology application and research, data resources, human resources, planning, standards, supervision, coordination, social participation, safety safeguards, market access, publicity, subsidies, financial support, government purchase of services, pilot projects, and user training.

The older adults’ need dimension was informed by the WHO Active Ageing Framework, which emphasises health, participation, and security, and was adapted to the smart health and older adult care policy context (10). Older adults’ needs were coded according to the beneficiary need, problem, or support domain addressed by each statement, rather than according to the policy instrument used to address it. First-level needs were classified as health-related needs, participation and development needs, and security-related needs. The second-level need codes were grouped under health-related needs (H), participation and development needs (P), and security-related needs (S). At the second level, 15 need categories were coded, covering daily care, medical and health services, safety, accessibility, rights protection, economic security, age-friendly environments, emotional and social support, participation, cultural and educational development, autonomous living, and value realisation.

The digital inclusion orientation dimension was developed with reference to digital inclusion and digital inequality research, which emphasises access, skills, affordability, safety, and meaningful participation in digital environments (3, 4, 6, 7). Digital inclusion orientations were coded according to whether and how each statement explicitly addressed older adults’ access to, capability to use, affordability of, protection in, or alternatives to digital health and care systems. Seven original categories were coded: no explicit digital inclusion orientation, digital access, digital skills, digital affordability, digital safety, offline alternatives, and age-friendly digital environment. This dimension captured whether and how policy statements addressed older adults’ access to, use of, and protection in digital health and care systems. The category “no explicit digital inclusion” did not mean that a statement lacked digital or care-related content; rather, it indicated that the statement did not explicitly address older adults’ digital access, skills, affordability, safety, offline alternatives, or age-friendly digital environments.

Because all three analytical dimensions draw on general theoretical frameworks originally developed in international contexts, an iterative pilot coding process was conducted using a subset of policy documents to verify whether these general categories could adequately capture the specific administrative expressions used in Chinese older adult care policy texts, and to adjust category definitions accordingly before full-corpus coding. Within the older adults’ need dimension, several initially broad categories derived from the WHO Active Ageing Framework were found to be insufficiently discriminating and were refined into more specific second-level categories once concrete policy content was reviewed. For example, statements initially coded under the general category “spiritual and cultural development” were refined into a distinct “cultural and educational development” category when the text explicitly referenced online universities for older adults (网上老年大学). Within the policy instrument dimension, the broad category “information sharing” was disaggregated into more specific subcategories—including “data resource development,” “supervision,” and “standards”—depending on whether the underlying statement emphasised database construction, performance monitoring, or procedural standardisation, respectively. Within the digital inclusion orientation dimension, the seven categories derived from international digital divide and digital inclusion literature (3, 4, 6, 7) were assessed against the pilot sample and found to correspond closely to Chinese administrative terms, such as age-friendly transformation initiatives (适老化改造) mapping onto age-friendly digital environment. Because these constructs demonstrated close conceptual correspondence with the source frameworks, the seven original categories were retained without structural modification; however, coders were instructed to base coding decisions on the administrative meaning of Chinese policy terms rather than on literal translation equivalence. These pilot-derived refinements were documented and incorporated into the coding manual prior to full-corpus coding.

For statistical analysis, detailed categories were aggregated into broader analytical variables to reduce sparse cells and improve interpretability. Aggregation followed four principles: theoretical consistency, semantic similarity, functional consistency, and statistical applicability. The 26 policy instrument codes were aggregated into six categories: fiscal support, service capacity, planning and demonstration, technology and data, platform interconnection, and regulation and safeguards. The 15 older adult need codes were aggregated into six categories: health care, service access and environment, rights and safety, economic support, social–emotional support, and development and autonomy. The seven digital inclusion codes were aggregated into three categories: no explicit digital inclusion orientation, access and environment, and capability-building and safety protections.

Detailed coding categories and operational definitions are provided in Supplementary Table S2, illustrative coding examples are provided in Supplementary Table S3, and the mapping from original codes to aggregated analytical variables is reported in Supplementary Table S4.

### 2.6 Coding procedure and reliability assessment

The coding team consisted of three authors. The first author designed the coding scheme, prepared the coding manual, organised coder training, supervised the coding process, and facilitated the resolution of ambiguous cases. The second and third authors, who had academic backgrounds in public administration/public service management and public health/health management, conducted the main coding work.

Following the coding scheme described above, the coders were trained using the coding manual and a pilot set of policy statements. During formal coding, each coding unit was assigned one dominant code for each analytical dimension. Ambiguous cases were flagged during coding and discussed within the research team before the final dataset was prepared for statistical analysis. The final dataset was produced after discussion and reconciliation of flagged cases.

To assess intercoder reliability, a random sample of 20% of the 1,033 coding units was independently coded by the second and third authors using the same coding manual. The reliability sample included 207 coding units. Cohen’s kappa was calculated based on the independent coding results before reconciliation to evaluate intercoder agreement for each coding dimension (17). The kappa values were 0.86 for first-level policy instruments, 0.79 for second-level policy instruments, 0.88 for first-level older adults’ need domains, 0.76 for second-level older adults’ need domains, and 0.81 for digital inclusion orientations. According to the criteria proposed by Landis and Koch, these values indicate substantial or almost perfect intercoder reliability (18). Disagreements were subsequently discussed between the two coders. When consensus could not be reached, the first author reviewed the case and made the final coding decision after discussion with the research team.

### 2.7 Statistical analysis

The analysis combined descriptive statistics, cross-tabulations, effect-size estimation, and residual diagnostics. Descriptive statistics were first used to summarise the distribution of coding units across the analytical dimensions and across the four policy stages. Cross-tabulations were then constructed to examine textual associations among policy stages, policy instruments, digital inclusion orientations, and older adults’ need domains. Aggregated variables were used in these analyses because some original categories contained small counts and were difficult to interpret in cross-tabulations. Aggregation also helped reduce sparse cells and improve the stability and readability of the tabulated results.

Associations between categorical variables were assessed using Pearson’s chi-square tests, and the strength of association was estimated using Cramer’s V (19). Because statistical significance can be influenced by sample size, Cramer’s V was interpreted together with percentage distributions and adjusted standardised residuals rather than relying on *p*-values alone. When chi-square tests indicated statistically significant associations, adjusted standardised residuals were used to identify the cells that deviated from expected frequencies. The adjusted standardised residual was calculated as follows:


$$AR_{\mathrm{ij}}=\frac{O_{\mathrm{ij}}-E_{\mathrm{ij}}}{\sqrt{E_{\mathrm{ij}}(1-r_{i})(1-c_{j})}}$$

where $O_{\mathrm{ij}}$ is the observed frequency, $E_{\mathrm{ij}}$ is the expected frequency under independence, $r_{i}$ is the row marginal proportion, and $c_{j}$ is the column marginal proportion. Values |AR| ≥ 1.96 were initially flagged as nominally higher or lower frequencies than expected at the 0.05 level. Given the relatively large number of cell-wise comparisons involved in the cross-tabulations, the Benjamini–Hochberg false discovery rate (FDR) procedure was additionally applied to reduce the risk of Type I errors arising from multiple comparisons (20). Cells that remained significant after FDR correction were interpreted as statistically more robust deviations, whereas cells that met only the uncorrected residual threshold were treated as descriptive patterns. These residuals were used as descriptive diagnostics to identify over-represented or under-represented textual combinations, rather than as evidence of causal relationships. Because multiple policy statements were extracted from the same policy documents, the independence assumption underlying Pearson’s chi-square tests may be partially violated. As a sensitivity check, we examined the distribution of coding units across documents and conducted supplementary Rao–Scott design-adjusted chi-square tests using policy document ID as the clustering unit. The 1,033 coded statements were clustered within 35 national policy documents, with each document contributing between 18 and 42 statements. The mean number of statements per document was 29.51 and the median was 30, indicating that no single document dominated the dataset. The original Pearson chi-square tests were retained to describe statement-level textual association patterns, while the Rao–Scott adjusted tests were used as sensitivity analyses to assess whether the main associations remained robust after accounting for within-document dependence.

Microsoft Excel was used to construct the coding table, manage coding records, and organise the final coded dataset. SPSS was used to generate cross-tabulations, conduct Pearson’s chi-square tests, calculate Cramer’s V, and obtain adjusted standardised residuals. R was used to produce bubble plots and heatmaps for visualising the cross-tabulation results. Because the analysis was based on counts of coded policy statements rather than individual-level observations, the statistical tests were used to describe association patterns among textual categories rather than to infer causal relationships or implementation effects. Complete cross-tabulation outputs are provided in Supplementary Table S5.

## 3 Results

### 3.1 Descriptive distribution of policy documents and coding dimensions

#### 3.1.1 Overview of the policy sample

Table 1 summarises the temporal distribution of the policy sample. The corpus covered 35 national-level documents and 1,033 valid coded policy statements issued between 2013 and 2025. The distribution was temporally uneven: the age-friendly deepening and silver economy application stages together accounted for 64.1% of all coded statements. This concentration suggests that smart health and older adult care received increased textual attention in national policy documents after 2020, particularly alongside age-friendly digital transformation policies and the subsequent silver economy agenda. This temporal unevenness should be considered when interpreting the stage-based comparisons reported below. Detailed information on document type, issuing authority, and document title is provided in Supplementary Table S1.

**Table 1:** Distribution of policy documents and coded policy statements by policy stages.

Policy stage	Period	Representative policy documents	No. of documents	Coded policy statements
Policy incubation	2013–2016	Several Opinions on Accelerating the Development of the Elderly Care Service Industry; Guiding Opinions on Actively Promoting the Internet Plus Action	7	197
Pilot access	2017–2019	Action Plan for the Development of the Smart Health and Elderly Care Industry (2017–2020)	6	174
Age-friendly deepening	2020–2023	Implementation Plan for Effectively Solving the Difficulties Faced by Older Adults in Using Smart Technologies; Action Plan for the Development of the Smart Health and Elderly Care Industry (2021–2025)	12	384
Silver economy application	2024–2025	Opinions on Developing the Silver Economy and Improving the Well-Being of Older Adults; Notice on Carrying out Pilot Projects for Intelligent Elderly Care Service Robots	10	278
Total	2013–2025	—	35	1,033

Policy stages were defined according to major policy milestones and thematic shifts in the smart health and older adult care policy agenda. Coded policy statements refer to valid coding units retained after segmentation and data cleaning.

#### 3.1.2 Distribution of aggregated coding dimensions

Table 2 presents the frequency distribution across the three aggregated analytical dimensions. The distributions reveal several notable asymmetries. Within the policy instrument dimension, service capacity and technology and data together accounted for more than half of all coded statements, whereas fiscal support accounted for a small proportion. Within the digital inclusion orientation dimension, nearly half of all statements carried no explicit digital inclusion orientation, and access and environment was more frequent than capability-building and safety protections. Within the older adults’ needs dimension, health care and service access and environment jointly accounted for more than 70% of statements, whereas social–emotional support and development and autonomy accounted for relatively small proportions.

**Table 2:** Descriptive distribution of aggregated analytical dimensions (*N* = 1,033).

Dimension	Category	*n*	%
Policy instruments	Fiscal support	51	4.9
	Service capacity	304	29.4
	Planning and demonstration	139	13.5
	Technology and data	230	22.3
	Platform interconnection	146	14.1
	Regulation and safeguards	163	15.8
Digital inclusion orientations	Access and environment	436	42.2
	Capability and protection	93	9
	No explicit orientation	504	48.8
Older adults’ need domains	Health care	360	34.8
	Service access and environment	369	35.7
	Rights and safety	167	16.2
	Economic support	42	4.1
	Social–emotional support	34	3.3
	Development and autonomy	61	5.9

### 3.2 Descriptive temporal patterns across policy stages

At the aggregated category level, statement-level Pearson chi-square tests showed associations between policy stage and all three analytical dimensions, with small effect sizes (Table 3). The robustness of these stage-related associations was further examined using document-cluster-adjusted sensitivity analysis.

**Table 3:** Chi-square tests of association between policy stages and aggregated analytical dimensions.

Cross-tabulated Dimensions	*χ* ^2^	df	*p*	Cramer’s V
Policy stage × Policy instruments	53.088	15	<0.001	0.131
Policy stage × Digital inclusion orientations	16.55	6	0.011	0.09
Policy stage × Older adults’ need domains	49.919	15	<0.001	0.127

All cells met the minimum expected count criterion (≥5). Tests are based on statement-level counts; document-cluster-adjusted sensitivity analyses are reported in Supplementary Table S6.

Table 4 further specifies these temporal patterns by reporting within-stage percentages and adjusted standardised residuals. For policy instruments, the stage-based distribution indicated differences in the relative emphasis placed on resource mobilisation, service expansion, technological instruments, and regulatory safeguards across policy stages. Fiscal support and service capacity were above expected in Stage 1, while fiscal support was below expected in Stage 3. Platform and interconnection instruments were above expected in Stage 2. Technology and data instruments accounted for larger shares in Stages 3 and 4, although their residuals did not exceed the corrected significance threshold. For older adults’ need domains, health care was above expected in Stage 1 and below expected in Stage 2, while service access and environment was above expected in Stage 2. Development and autonomy, although relatively infrequent overall, was above expected in Stage 3. Economic support showed a non-linear pattern, with a negative residual in Stage 3 and a positive residual in Stage 4 that did not remain significant after FDR correction. For digital inclusion orientations, no explicit digital inclusion orientation was over-represented in Stage 1 and under-represented in Stage 3, whereas capability and protection was over-represented in Stage 3. Access and environment remained the most frequent explicit digital inclusion orientation across all four stages.

**Table 4:** Distribution of coding dimensions across policy stages with FDR-corrected residual diagnostics.

Dimension	Category	Stage 1 *n* = 197	Stage 2 *n* = 174	Stage 3 *n* = 384	Stage 4 *n* = 278
Policy instruments	Fiscal support	**8.6 (+2.7)**	2.3 (−1.8)	**2.3 (−3.0)**	7.6 (+2.4)^†^
	Service capacity	**38.1 (+3.0)**	23.6 (−1.9)	29.9 (+0.3)	26.3 (−1.4)
	Planning and demonstration	14.7 (+0.6)	19.0 (+2.3)^†^	10.4 (−2.2)^†^	13.3 (−0.1)
	Technology and data	15.7 (−2.4)^†^	18.4 (−1.3)	25.0 (+1.6)	25.5 (+1.5)
	Platform interconnection	12.2 (−0.9)	**20.7 (+2.7)**	12.8 (−1.0)	13.3 (−0.5)
	Regulation and safeguards	10.7 (−2.2)^†^	16.1 (+0.1)	19.5 (+2.5)^†^	14.0 (−0.9)
Digital inclusion orientations	Capability and protection	5.1 (−2.1)^†^	6.9 (−1.1)	**12.2 (+2.8)**	8.6 (−0.3)
	Access and environment	37.6 (−1.5)	47.1 (+1.4)	43.8 (+0.8)	40.3 (−0.8)
	No explicit orientation	**57.4 (+2.7)**	46.0 (−0.8)	44.0 (−2.4)^†^	51.1 (+0.9)
Older adults’ need domains	Rights and safety	11.2 (−2.1)^†^	16.1 (+0.0)	19.0 (+1.9)	15.8 (−0.2)
	Development and autonomy	2.5 (−2.2)^†^	3.4 (−1.5)	**9.4 (+3.6)**	5.0 (−0.7)
	Service access and environment	33.0 (−0.9)	**46.6 (+3.3)**	32.8 (−1.5)	34.9 (−0.3)
	Health care	**47.2 (+4.0)**	**25.9 (−2.7)**	33.1 (−0.9)	34.2 (−0.3)
	Economic support	4.6 (+0.4)	5.2 (+0.8)	**1.8 (−2.8)**	6.1 (+2.0)^†^
	Social–emotional support	1.5 (−1.5)	2.9 (−0.3)	3.9 (+0.9)	4.0 (+0.7)

Values are percentages of policy units within each stage, with adjusted residuals shown in parentheses. Stage 1, policy incubation; Stage 2, pilot access; Stage 3, age-friendly deepening; Stage 4, silver economy application. Because this table involves 60 cell-wise residual diagnostics, the Benjamini–Hochberg false discovery rate (FDR) procedure was applied to control for multiple comparisons. Bold values indicate cells that remained statistically significant after FDR correction at *q* < 0.05. Values marked with ^†^met the conventional uncorrected residual threshold of |AR| ≥ 1.96 but did not remain significant after FDR correction; these cells are interpreted as descriptive patterns rather than statistically robust deviations. AR, adjusted standardised residual. Document-cluster-adjusted sensitivity analyses are reported in Supplementary Table S6.

### 3.3 Co-occurrence patterns across analytical dimensions

The pairwise chi-square tests reported in Table 5 showed statistically significant associations among policy instruments, older adults’ need domains, and digital inclusion orientations. These results indicate non-random co-occurrence patterns among the coded textual categories.

**Table 5:** Pairwise chi-square tests among policy instruments, older adults’ need domains, and digital inclusion orientations.

Dimension 1	Dimension 2	*df*	*χ* ^2^	*p*	Cramer’s *V*
Policy instruments	Older adults’ need domains	25	697.422	< 0.001	0.367
Policy instruments	Digital inclusion orientations	10	290.000	< 0.001	0.375
Older adults’ need domains	Digital inclusion orientations	10	164.171	< 0.001	0.282

Effect size labels are used descriptively. As a rough guide, Cramer’s *V* values around 0.10, 0.30, and 0.50 are often interpreted as small, medium, and large associations, respectively, although interpretation depends on table size and substantive context. Tests are based on 1,033 coded policy statements.

Instrument–need configurations. The cross-tabulation of policy instrument categories and older adults’ need domains yielded a statistically significant association, *χ*^2^(25) = 697.422, *p* < 0.001, Cramer’s *V* = 0.367 (Table 5; Figure 3; Supplementary Table S5). This pattern points to a clear functional specialisation within the coded policy sample. Several instrument types were over-represented in specific need domains: service capacity instruments in health care (AR = +9.3), planning and demonstration and platform interconnection instruments in service access and environment (AR = +8.2 and +7.6, respectively), regulation and safeguards instruments in rights and safety (AR = +15.0), and fiscal support instruments in economic support (AR = +14.5). As shown in Figure 3, the strongest positive residuals were concentrated in a small number of functionally specialised cells, suggesting that particular instruments tended to co-occur with particular need domains. Service capacity instruments were the only category that also showed significant over-representation in development and autonomy (AR = +6.1). By contrast, regulation and safeguards instruments were significantly under-represented in development and autonomy (AR = −3.5), suggesting that this instrument type appeared less frequently in policy statements addressing older adults’ higher-order developmental needs.

**Figure 3:**
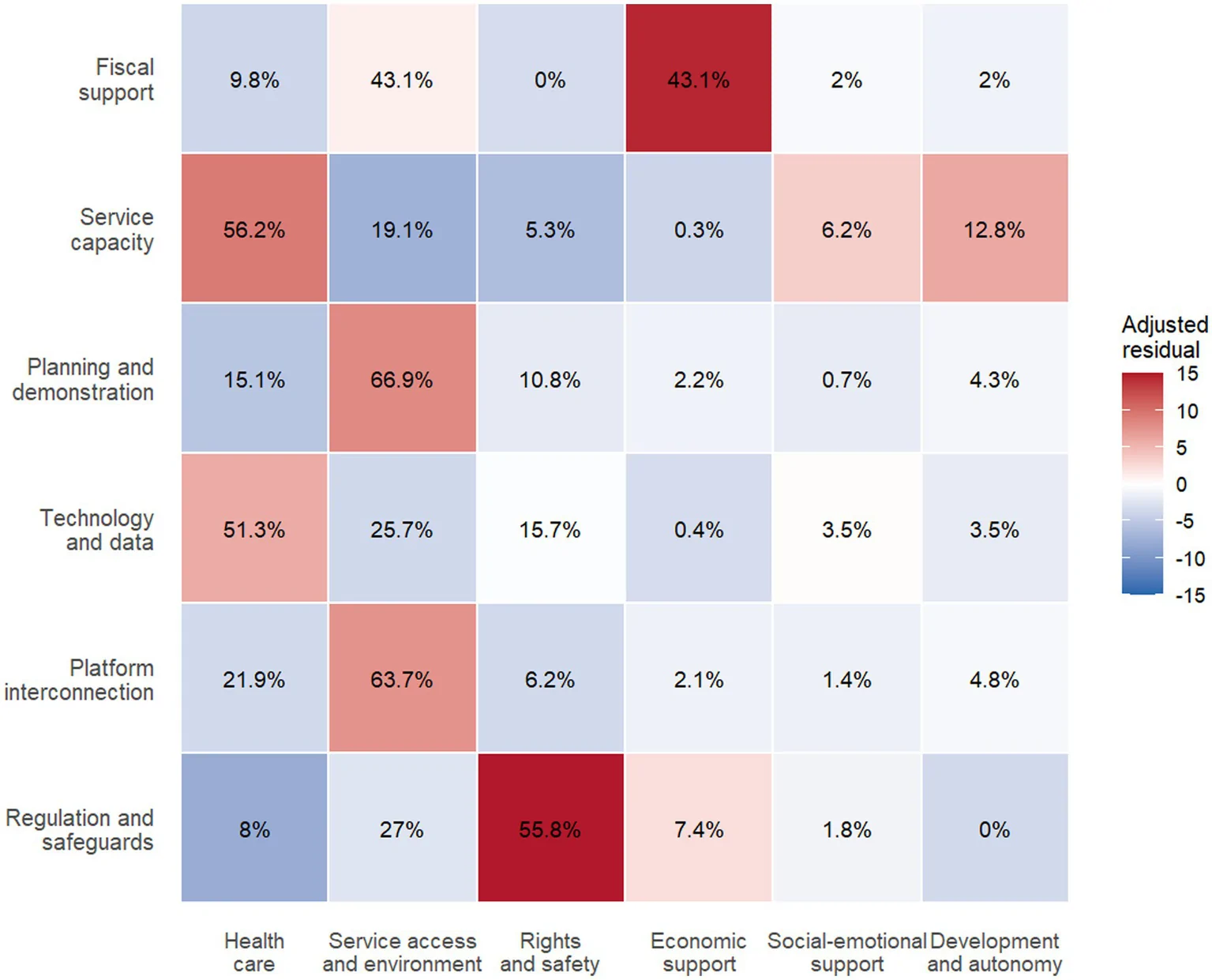
Co-occurrence between policy instruments and older adults’ need domains. Cell labels indicate row percentages within each policy instrument category. Colour represents adjusted standardised residuals, with red indicating over-representation and blue indicating under-representation relative to expected frequencies under independence. Stronger colour intensity indicates larger residual magnitude. Adjusted standardised residuals with absolute values ≥ 1.96 indicate statistically significant deviations at *p* < 0.05, two-tailed.

Instrument–digital inclusion orientation configurations. The cross-tabulation of policy instrument categories and digital inclusion orientations produced the largest Cramer’s V among the three pairwise tests, *χ*^2^(10) = 290.000, *p* < 0.001, Cramer’s *V* = 0.375, indicating a moderate association (Table 5; Figure 4; Supplementary Table S5). Digital inclusion orientations were not evenly distributed across instrument types. Technology and data instruments and platform interconnection instruments were significantly over-represented in access and environment (AR = +10.4 and +8.9, respectively), consistent with the infrastructure- and connectivity-oriented nature of these instruments. Regulation and safeguards instruments were significantly over-represented in no explicit digital inclusion orientation (AR = +6.4), suggesting that regulatory provisions often addressed governance and protection issues without an explicit digital inclusion orientation. Fiscal support instruments showed significant under-representation in access and environment (AR = −6.3) and significant over-representation in capability-building and safety protections (AR = +6.2), although this pattern should be interpreted cautiously given the small number of fiscal support statements. Service capacity instruments were also significantly over-represented in no explicit digital inclusion orientation (AR = +4.5), suggesting that many service-related provisions addressed older adults’ needs without explicitly framing them as digital inclusion issues.

**Figure 4:**
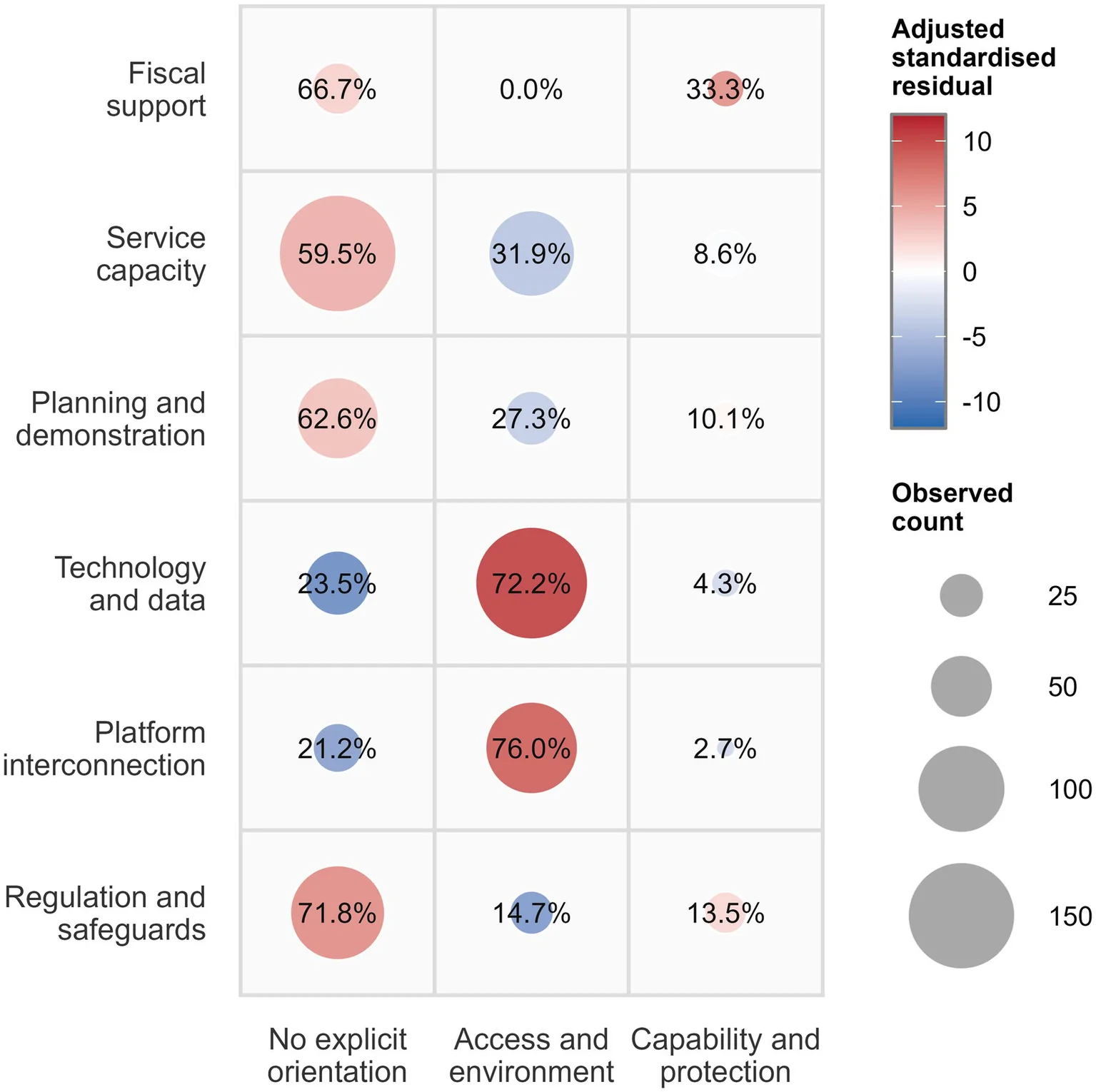
Co-occurrence between policy instruments and digital inclusion orientations. Cell labels indicate row percentages within each policy instrument category. Bubble size represents cell count. Colour represents adjusted standardised residuals, with red indicating over-representation and blue indicating under-representation relative to expected frequencies under independence. Stronger colour intensity indicates larger residual magnitude. Adjusted standardised residuals with absolute values ≥ 1.96 indicate statistically significant deviations at *p* < 0.05, two-tailed. For readability, “No explicit orientation” refers to no explicit digital inclusion orientation, and “Capability and protection” refers to capability-building and safety protections.

Need domain–digital inclusion orientation configurations. The cross-tabulation of older adults’ need domains and digital inclusion orientations yielded a statistically significant association, *χ*^2^(10) = 164.171, *p* < 0.001, Cramer’s *V* = 0.282 (Table 5; Figure 5; Supplementary Table S5). Service access and environment showed significant over-representation in access and environment (AR = +4.0), and health care was also over-represented in this digital inclusion orientation (AR = +2.7). By contrast, rights and safety, economic support, and development and autonomy were significantly over-represented in capability-building and safety protections (AR = +6.5, +5.1, and +6.7, respectively). Social–emotional support showed no significant adjusted residuals across digital inclusion orientations, suggesting that policy statements related to emotional companionship, social participation, community belonging, and intergenerational connection were not consistently framed through a distinctive digital inclusion orientation. Across the three pairwise tests, the associations involving policy instruments had the largest Cramer’s V values, suggesting that instrument type was the most structured dimension in the observed co-occurrence patterns.

**Figure 5:**
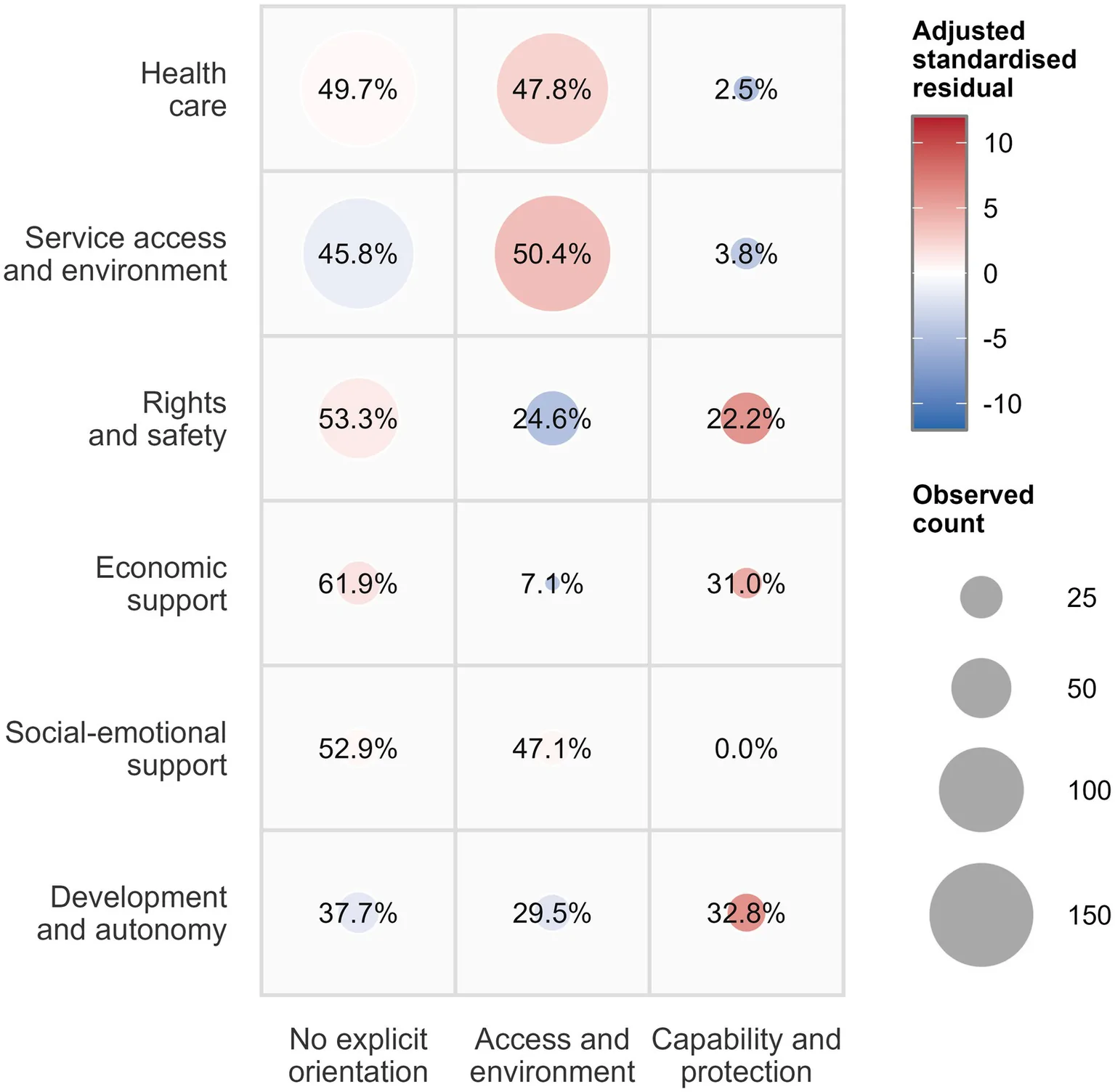
Co-occurrence between older adults’ need domains and digital inclusion orientations. Cell labels indicate row percentages within each older adults’ need domain. Bubble size represents cell count. Colour represents adjusted standardised residuals, with red indicating over-representation and blue indicating under-representation relative to expected frequencies under independence. Stronger colour intensity indicates larger residual magnitude. Adjusted standardised residuals with absolute values ≥ 1.96 indicate statistically significant deviations at *p* < 0.05, two-tailed. For readability, “No explicit orientation” refers to no explicit digital inclusion orientation, and “Capability and protection” refers to capability-building and safety protections.

As a sensitivity analysis, Rao–Scott design-adjusted chi-square tests were conducted using policy document ID as the clustering unit. The three cross-dimensional associations remained statistically significant after document-cluster adjustment, whereas the stage-related associations were attenuated and no longer statistically significant. Full results are reported in Supplementary Table S6.

## 4 Discussion

### 4.1 Access-oriented framing and the capability gap

At the level of national policy design, China’s smart health and older adult care documents framed digital inclusion primarily through an access-and-environment logic. Technology and data instruments and platform interconnection instruments were strongly associated with access- and environment-oriented inclusion, while capability-building and protection-oriented measures were comparatively less prominent. This pattern is consistent with prior analyses of China’s smart older adult care and digital ageing policies, which have documented a development logic centred on infrastructure construction, platform building, pilot demonstration, and technology-enabled service provision (14, 21, 22). It also aligns with a broader international tendency for digital health strategies to prioritise infrastructure deployment, electronic health record systems, and telemedicine platforms as foundational conditions for digital transformation (23).

However, these textual patterns suggest a potential gap between this access-based framing and the multidimensional conditions required for equitable digital participation. Van Dijk’s three-level digital divide framework provides a useful theoretical basis for interpreting this gap (3). Physical access—the first-level divide—is necessary but insufficient for digital inclusion. The second-level divide, rooted in disparities in digital skills and usage capabilities, and the third-level divide, concerning unequal capacities to translate digital engagement into meaningful health and social outcomes, represent equally consequential barriers (3–5). In health and older adult care settings, these higher-order divides are particularly important: older adults may remain at risk of practical exclusion even when digital services are formally available if they lack affordable access, digital literacy, timely support, confidence, or safe service conditions (6, 7, 12, 24). In this sense, offline alternatives should be understood not as digital inclusion itself, but as safety-net mechanisms that help prevent exclusion under increasingly digitalised service delivery (25, 26).

Recent empirical evidence reinforces the relevance of this concern, while also underscoring the need to distinguish policy design from implementation outcomes. Studies in China have linked digital divide and digital health divide indicators to health inequalities and health-related outcomes among older adults (27–29). Other research further indicates that the benefits of digital economy development are unevenly distributed, with older adults living in larger cities and those with higher human, physical, and digital literacy capital being better positioned to capture digital dividends (13, 30, 31). Although the present study does not measure individual-level service use or health outcomes, this literature suggests that the limited textual emphasis on capability-building and protection in national policy framing may indicate a design-level mismatch between national policy framing and the empirically established determinants of digital health equity.

Accordingly, the main implication should be understood as a design-level concern rather than an evaluation of implementation performance. When national policy texts place greater emphasis on digital access, platforms, and data systems than on capability-building, protections, and fallback arrangements, digital health and care services may become formally more available while remaining practically difficult for some older adults to use. This risk is particularly relevant for older adults with limited digital skills, financial resources, confidence, or social support. The issue, therefore, is not simply whether digital services are supplied, but whether policy design also specifies the user-side conditions required to access, understand, trust, and benefit from them.

### 4.2 Policy instrument inertia and the limits of incremental inclusion

The temporal analysis showed modest descriptive differences across policy stages. At the statement level, policy stage was associated with policy instruments, digital inclusion orientations, and older adults’ need domains, but the corresponding effect sizes were small and the associations were attenuated after document-cluster adjustment. Accordingly, the temporal results are treated here as descriptive patterns rather than as the primary inferential basis of the study. These patterns point to incremental rebalancing in policy attention rather than a fundamental reconfiguration of the policy instrument mix. Across stages, the national-level policy architecture remained largely dominated by supply-side service, technology, and platform-oriented instruments.

This pattern warrants theoretical attention because it is consistent with policy instrument research showing that policy instrument mixes often exhibit inertia and path dependence even when overarching policy goals evolve (9, 27). Once established, supply-side instrument configurations may persist because they are embedded in institutional routines, bureaucratic capacities, and existing governance arrangements that are difficult to reorganize (9, 27, 28). In the present case, the descriptive temporal patterns suggest that the increasing visibility of digital inclusion in national policy texts did not displace the existing supply-side and technology-oriented policy architecture. Instead, inclusion-related content appears to have been incorporated into the existing instrument mix through partial adjustment and supplementation.

Mahoney and Thelen’s concept of institutional layering offers a useful theoretical explanation for this dynamic (28). In layering, new policy elements are added alongside existing institutional arrangements without necessarily replacing them. The present findings are consistent with this mechanism: digital inclusion goals appear to have been layered onto a dominant access- and technology-oriented policy architecture rather than reorganising the framework itself. The underlying logic of access provision remained visible as the institutional core, while capability-building, protection, and fallback arrangements were incorporated as supplementary content within instruments whose fundamental orientation appeared largely unchanged. The resulting national-level policy architecture may therefore be characterised as formally more inclusive but only incrementally transformed.

The stage-specific distributions provide descriptive support for this incremental pattern. In the age-friendly deepening stage (2020–2023), which overlapped with the COVID-19 pandemic and the widespread use of health codes and other digital access requirements, capability and protection accounted for a larger share of coded statements than in earlier stages. However, they still represented only 12.2% of coded statements in this stage, whereas access and environment and no explicit inclusion together accounted for 87.8%. Similarly, the silver economy application stage (2024–2025) showed a relative increase in economic support, but this pattern should be read cautiously given the short duration of the stage and the attenuation of stage-related associations after document-cluster adjustment. Overall, the temporal patterns suggest content supplementation rather than displacement: new inclusion-oriented concerns appeared to be added to an existing access- and technology-oriented policy architecture rather than reorganising it. The study’s central claims are therefore based primarily on the cross-dimensional associations reported in Table 5, which remained statistically significant after document-cluster adjustment, as shown in Supplementary Table S6.

### 4.3 Selective alignment across need domains and its implications for healthy ageing

The association analyses revealed a selectively aligned national-level policy structure with limited attention to participatory dimensions of healthy ageing. Service access and environment and health care needs—which together accounted for more than 70% of all coded policy statements—were more strongly associated with access-and-environment digital inclusion orientations. This pattern suggests that digital technologies were predominantly framed as tools for improving service delivery, health management, and monitoring systems. By contrast, rights and safety, development and autonomy, and economic support needs were more closely associated with capability and protection, but these categories accounted for a smaller share of policy content. Most notably, social–emotional support showed no significant residual pattern across digital inclusion orientations, suggesting that digital inclusion was not systematically articulated in relation to emotional companionship, social participation, community belonging, or intergenerational connection.

A related asymmetry appeared in the instrument–need configuration. Regulation and safeguards instruments were strongly associated with rights and safety needs (AR = +15.0) but significantly under-represented in development and autonomy needs (AR = −3.5). This pattern suggests that the regulatory apparatus was framed more around protection and compliance—such as data security, fraud prevention, and market oversight—than around enabling older adults’ learning, autonomous choice, or social contribution. Within this policy corpus, regulation therefore appears to be articulated more as a means of governing risk than as a means of enabling agency.

This pattern is theoretically significant from a healthy ageing perspective. The WHO Active Ageing Policy Framework and the Decade of Healthy Ageing emphasise that well-being in later life depends not only on disease management and service access, but also on participation, security, autonomy, and enabling environments (10, 29, 30). From this perspective, the present findings suggest that digital inclusion in China’s national smart health and older adult care policy texts is most systematically articulated in relation to the health care and service-access dimensions of ageing. Security-related concerns are partially addressed through regulatory and fiscal instruments, but the participation dimension receives much weaker attention, particularly in relation to social–emotional support, developmental opportunities, and older adults’ agency.

The potential implications of this selective alignment are supported by recent longitudinal evidence from China. Dong and Chen (31), drawing on five waves of CHARLS data, showed that the three levels of the digital divide operate as a chain: access inequalities are linked to usage inequalities, which in turn are associated with unequal outcomes in successful ageing, including physical functioning, cognitive health, and social engagement. Crucially, this chain effect was moderated by education, urban residence, and socioeconomic status, suggesting that the conversion of digital access into successful ageing outcomes may be more favourable for socioeconomically advantaged older adults. Duan similarly found that digital economy development had heterogeneous effects across subgroups, with higher-educated, urban, and higher-income older adults capturing a disproportionate share of the digital dividend (32). Together, these studies point to a risk of widening inequalities in successful ageing: supply-side digital policy designs that concentrate on access and service delivery, without sufficient attention to capability-building and social participation, may disproportionately benefit already advantaged older adults while leaving more vulnerable groups behind (6, 7, 31, 32).

The differentiated need–instrument association pattern identified in Section 3.3 further illuminates this problem. Development and autonomy and social–emotional support needs were most often linked to service capacity instruments in the coded policy statements, a category in which explicit digital inclusion was often absent. This points to a twofold design-level mismatch: the need domains most relevant to participation-oriented healthy ageing were associated with instruments that are less likely to incorporate explicit digital inclusion. Together with prior evidence of fragmented digital ageing policy formation in China (22), this finding suggests that the participation-pillar deficit identified here may reflect a recurring pattern in current national policy design rather than an isolated coding result. Because social participation, autonomy, and enabling environments are central components of active and healthy ageing (10, 29, 30), the weak textual embedding of digital inclusion in social–emotional and development-oriented need domains represents not only a policy design gap but also a missed opportunity to support more equitable digital ageing. If supply-side digital health and care systems are not complemented by capability-building and participatory support, they may widen inequalities in older adults’ ability to benefit from these systems (6, 7, 31–34).

These interpretations should nevertheless be situated within the evidentiary scope of this study. The analysis identifies patterns of national-level policy design and textual prioritisation, rather than implementation outcomes, local adaptations, or older adults’ lived experiences of digital health and care. National policy documents are analytically important because they define overarching policy objectives, allocate administrative attention, and set the agenda for subsequent local implementation; however, they cannot fully capture how digital inclusion is operationalised across provincial, municipal, and grassroots contexts. The access-oriented pattern identified here should therefore be understood as a design-level orientation in national policy texts, not as evidence that older adults have achieved equitable access to, or meaningful use of, digital health and care services in practice. From a health equity perspective, this distinction is particularly important, because rural older adults, low-income groups, people with disabilities, the oldest-old, and those with limited digital literacy may face barriers that are not fully visible in national-level policy wording (6, 7, 35, 36). Future research should triangulate national policy-text analysis with local policy documents, administrative data, and field-based evidence to examine whether and how these design-level patterns translate into unequal implementation and ageing outcomes.

### 4.4 Policy implications

Several policy implications follow from these design-level patterns, but they should be read within the evidentiary scope of this study. The analysis identifies patterns of national-level policy design and textual prioritisation, rather than implementation outcomes, local adaptations, or older adults’ lived experiences of digital health and care. The dominant instrument configuration—technology deployment, platform construction, and service capacity expansion—indicates a policy orientation that extends the reach of digital health and care services without systematically addressing the conditions under which older adults can meaningfully access, use, and benefit from them. Closing this gap requires not simply more investment in the same instrument categories, but a deliberate redistribution of policy attention toward demand-side support, capability-building, inclusive safeguards, and equity-oriented implementation mechanisms.

A central implication is that capability and affordability should be treated as core design requirements rather than supplementary add-ons. In the coded policy statements, digital literacy support and cost-reduction measures were more often associated with fiscal support and regulation and safeguards instruments, which together accounted for only 20.7% of all policy statements, while the dominant supply-side instruments were less explicitly linked to user-side conditions. A more coherent instrument mix would embed capability and affordability provisions directly within technology deployment, platform construction, and service rollout policies. In practical terms, this would require age-friendly interface standards, accessible service channels, and co-located digital literacy support to be incorporated into procurement, platform evaluation, and service accreditation processes. For fiscal support instruments specifically, policy attention should shift beyond institution-oriented procurement toward individual-level barriers, including subsidised internet access, age-friendly device programmes, and sustained community-based training, with explicit priority for rural, low-income, and low-education older adults who are less likely to benefit from infrastructure expansion alone. Such measures would require coordinated implementation by health commissions, civil affairs departments, finance agencies, community service organisations, and technology providers, with responsibilities specified across procurement, training delivery, subsidy allocation, and service monitoring.

A parallel implication concerns the scope of regulation and safeguards instruments. In the coded policy statements, these instruments were disproportionately anchored to rights and safety needs—such as data protection, fraud prevention, and market oversight—while social–emotional support and development and autonomy needs received much weaker regulatory grounding. This pattern points not only to a distributional imbalance, but also to a relatively narrow textual conception of regulation in digital ageing governance. Regulatory obligations should therefore extend beyond risk control to include mandatory age-friendly design standards for public-facing health applications, enforceable requirements for maintaining offline service channels, and accountability mechanisms for algorithmic systems that may disadvantage older users (7, 25, 26). In this framework, offline alternatives should be understood as safety-net or fallback arrangements for equitable digital inclusion, rather than as digital inclusion itself (25, 26). Their maintenance should be recognised as a binding service-continuity obligation rather than a temporary transitional measure, because the removal of non-digital channels may disproportionately affect older adults with the lowest digital capabilities—precisely those whom digital inclusion policies are intended to support. Operationally, this would require health authorities, civil affairs departments, market regulators, and digital service providers to specify minimum offline-access standards, complaint-handling procedures, algorithmic audit requirements, and age-friendly usability indicators in procurement and service accreditation.

Underlying these specific recommendations is a more fundamental reframing. The present findings suggest that, at the level of national policy design, digital inclusion has been articulated primarily as an infrastructural and service-delivery problem—one to be addressed through platform expansion and service digitalisation. Yet the evidence discussed in this study points in a different direction: digital skills, affordability, and social participation should be understood as independent conditions shaping equitable healthy ageing, rather than as automatic downstream effects of access (3–7). Recognising digital inclusion as a social determinant of healthy ageing—rather than merely a technical accessibility requirement—would provide the normative foundation for the instrument reorientation described above (6, 7, 33). It would also help align national digital ageing policy design more closely with the participation and security pillars of the WHO Active Ageing Framework, which appear to be only partially addressed in the current policy corpus (10).

### 4.5 Limitations and future research

Several methodological limitations should be acknowledged. First, this study analyses policy texts and therefore examines national-level design intent rather than implementation processes or policy outcomes. The gap between policy design and policy effects is well recognised in policy studies and instrument analysis (9, 27, 37). The associations identified in this study should therefore be interpreted as patterns of policy content emphasis rather than as evidence of causal effects, implementation intensity, or actual service performance. Future research should combine text analysis with implementation evaluations, administrative data, budgetary information, or field-based evidence to assess whether the design-level gaps identified here are associated with real-world exclusion or unequal healthy ageing outcomes. Second, although intercoder reliability was satisfactory, with Cohen’s kappa ranging from 0.76 to 0.88, coding policy texts involves interpretive judgement that cannot be fully eliminated (15, 16). The single-dominant-code strategy, while necessary for categorical analysis, may not fully capture policy statements that simultaneously address multiple instruments, needs, and digital inclusion orientations. Because the unit of analysis was the coded policy statement rather than the policy document as a whole, the findings should be interpreted as patterns in policy content emphasis rather than as direct measures of policy implementation, administrative commitment, or resource allocation. To account for the non-independence of statements nested within policy documents, supplementary Rao–Scott design-adjusted chi-square tests were conducted using policy document ID as the clustering unit; these analyses supported the robustness of the main cross-dimensional associations, while stage-related associations were attenuated.

The study’s scope also warrants caution in generalization. The analysis is confined to national-level documents, and local governments may interpret, adapt, or supplement national directions in ways not captured here, including through implementation rules, funding arrangements, pilot programs, or offline service provisions. The degree to which digital inclusion is mainstreamed at provincial and municipal levels therefore remains an important empirical question. The stage-based analysis also requires cautious interpretation, as periodization decisions and the uneven distribution of documents across stages may affect the reading of temporal patterns, although the study’s core cross-sectional findings reported in Table 5 remained robust in document-cluster-adjusted sensitivity analyses, as reported in Supplementary Table S6. Beyond China, the Policy Alignment Framework developed in this study has yet to be tested in comparative contexts. Extending the framework across countries, welfare regimes, policy sectors, or levels of government would help identify transferable design principles and situate China’s experience within the broader global challenge of digital equity in ageing societies. Future studies should also incorporate subgroup-level evidence on rural older adults, low-income groups, people with disabilities, the oldest-old, and those with limited digital literacy to better assess the health equity implications of digital inclusion policies.

## 5 Conclusion

This study examined how digital inclusion is embedded in China’s national smart health and older adult care policies through a quantitative content analysis of 35 policy documents and 1,033 coded policy statements. The findings indicate an access-oriented but unevenly embedded model of digital inclusion in national policy texts. Technology and platform instruments were strongly associated with access- and environment-oriented inclusion, whereas capability-building, affordability, safety protections, and offline fallback arrangements remain concentrated in narrower fiscal and regulatory channels and were less clearly mainstreamed across the dominant service and technology instruments.

The analysis also shows that policy alignment varies across older adults’ needs. Health care and service access needs are relatively well represented, while social–emotional support and development and autonomy receive limited textual attention and are only weakly linked to digital inclusion. This pattern suggests that, at the level of national policy design, digital inclusion in smart health and older adult care policy is still articulated primarily as an infrastructural and service-delivery issue rather than as a multidimensional condition for capability, equity, and participation in later life.

The Policy Alignment Framework developed in this study offers a replicable tool for diagnosing such design-level gaps across policy systems. For China and other ageing societies undergoing digital transformation, equitable digital ageing is likely to require not only expanding smart health and care services, but also recalibrating policy instruments so that older adults can access, understand, afford, trust, and meaningfully use them.

## Data Availability

The policy documents analyzed are publicly available from official Chinese government sources. The coded dataset is available from the corresponding author upon reasonable request.
